# Root Plasticity and Elemental Stoichiometry Are Associated with Competitive Shifts Between *Azolla* and *Lemna* Under Different Nitrogen Levels

**DOI:** 10.3390/plants15121853

**Published:** 2026-06-15

**Authors:** Si Liu, Xiaoyue Liang, Yingcan Chen, Meijuan Li, Wenjing Li, Jiaen Zhang, Ronghua Li

**Affiliations:** 1 College of Natural Resources and the Environment, South China Agricultural University, Guangzhou 510642, China; liusi.scau.edu.cn@stu.scau.edu.cn (S.L.); liangxy56@stu.scau.edu.cn (X.L.); chenyingcan@stu.scau.edu.cn (Y.C.); 20253138049@stu.scau.edu.cn (W.L.); 2 Rice Research Institute, Guangdong Academy of Agricultural Sciences, Guangzhou 510640, China; limeijuan@gdaas.cn; 3 Guangdong Engineering Technology Research Centre of Modern Eco-Agriculture and Circular Agriculture, Guangzhou 510642, China; 4 Key Laboratory of Agro-Environment in the Tropics, Ministry of Agriculture and Rural Affairs, Guangzhou 510642, China

**Keywords:** nitrogen availability, interspecific competition, stoichiometric homeostasis, root plasticity, floating macrophytes

## Abstract

Nitrogen (N) availability fundamentally shapes the community structure and competitive dynamics of floating macrophytes in paddy ecosystems. This study investigated the competitive interactions between *Azolla* and *Lemna* by applying a gradient of N concentrations (0–12 mg L^−1^) across two experimental periods (November–January and March–May). Our results demonstrate a clear divergence in resource-use strategies between the two species: *Azolla* exhibited stronger stoichiometric homeostasis and a more conservative growth profile, retaining a competitive advantage under N-limiting conditions. Conversely, *Lemna* displayed a more opportunistic strategy, gaining a competitive advantage in N-rich environments through greater morphological plasticity and luxury nutrient uptake. This nitrogen-driven shift in competitive balance was associated with differences in root traits and stoichiometric flexibility. Stoichiometrically, *Lemna* exhibited greater flexibility in nutrient balance, including higher phosphorus accumulation under N-rich conditions, which may support rapid biomass expansion. Differences between the two experimental periods were also associated with variation in trait expression, suggesting that temporal context influenced how the two species responded to N enrichment. These findings highlight the importance of nitrogen management in steering floating-plant communities in paddy ecosystems: low-N inputs may help maintain *Azolla*-dominated communities with biofertilizer potential, whereas high-N conditions may favor *Lemna* and its rapid nutrient uptake.

## 1. Introduction

Nitrogen (N) is a major limiting nutrient in rice production, and high yields in modern paddy systems still depend heavily on synthetic N fertilizer [1]. Although this practice has supported rice productivity, it is often accompanied by low N use efficiency and increasing production costs [2]. Reducing fertilizer dependence by strengthening biological N fixation and internal N cycling is therefore important for sustainable rice production [3,4]. *Azolla*, a free-floating fern that forms a symbiosis with N-fixing cyanobacteria, has long been used as a biological N source in paddy fields [5]. Because it can rapidly accumulate N-rich biomass on the water surface and subsequently be incorporated into the soil, *Azolla* can contribute substantially to crop N demand and partially substitute for chemical N fertilizer [6].

In paddy fields, however, *Azolla* rarely grows alone. Small free-floating macrophytes commonly co-occur on the water surface, including *Lemna*, *Spirodela*, *Landoltia*, and *Wolffia*, among which *Lemna* is particularly common in rice fields and irrigation ditches [7]. Unlike *Azolla*, *Lemna* does not fix atmospheric N but relies on dissolved inorganic N in the surrounding water. Owing to its rapid clonal propagation, *Lemna* can quickly form dense mats under relatively high N availability [8]. Because two-dimensional physical space (water surface area) is a key limiting resource for floating plants, varying nutrient levels can fundamentally alter the intensity of their spatial competition [9]. We selected *Lemna* as the focal competitor in this study because it is one of the most widespread and ecologically representative non-diazotrophic floating macrophytes in paddy ecosystems, and it frequently shares the same water surface with *Azolla*. Comparing *Azolla* with *Lemna* is ecologically meaningful because it contrasts two fundamentally different nitrogen-acquisition strategies: symbiotic nitrogen fixation versus direct uptake of dissolved inorganic nitrogen. These contrasting resource-use strategies imply a fundamental trade-off: *Azolla* has a distinct advantage under N-deficient conditions, whereas *Lemna* responds more aggressively to N enrichment [10]. Consequently, the prevailing N status is generally assumed to largely determine their competitive balance. However, field observations frequently contradict this simple nutrient-driven model. Although *Azolla* can rapidly outgrow and suppress *Lemna* under optimal conditions, field monitoring shows that *Lemna* often remains more abundant in natural ponds [11]. Furthermore, their natural distributions exhibit weak correlations with bulk field nutrient levels [11]. This discrepancy suggests that actual community dominance is not solely governed by static N concentrations but may also be influenced by additional environmental factors. Yet, it remains unclear how variation in N availability, together with differences between environmental conditions, is associated with their competitive interactions, particularly in relation to submerged root foraging and stoichiometric strategies.

Functional traits, such as root morphology and C:N:P stoichiometry, directly reflect how these plants manage resources [12,13]. In aquatic plants, stoichiometric ratios provide an integrated indicator of growth performance because they describe the balance between carbon assimilation and nutrient investment [14]. For example, a lower C:N ratio generally indicates greater nitrogen allocation to proteins and photosynthetic machinery, which is often associated with faster biomass accumulation, whereas variation in N:P ratio reflects shifts in relative nitrogen versus phosphorus limitation and therefore the capacity to sustain growth under changing nutrient supply [15]. Since free-floating macrophytes rely on roots for spatial foraging, changes in N supply and environmental conditions strongly regulate root elongation and proliferation, driving shifts in biomass allocation between fronds and roots [16,17,18]. Concurrently, external N availability triggers distinct stoichiometric responses. For the diazotrophic *Azolla*, fluctuating N levels alter the balance between N_2_ fixation and direct N uptake, which requires homeostatic regulation [19,20]. *Lemna*, in contrast, typically engages in luxury nutrient consumption under enriched conditions, leading to elevated N:P ratios that fuel its rapid growth [21,22]. Comparing these morphological and stoichiometric adjustments therefore provides a useful basis for understanding differences in their competitive performance.

While the biofertilizer potential of *Azolla* and the nutrient recovery capacity of *Lemna* have been studied extensively in isolation, their competitive interactions remain poorly understood. Specifically, it is unclear how external N levels and differences between experimental periods are associated with their coexistence. To address this, we conducted a microcosm experiment across two experimental periods (November–January and March–May), subjecting *Azolla* and *Lemna* to four N concentrations (0, 4, 8, and 12 mg L^−1^) under monoculture and mixed-culture conditions. We hypothesized that the two species would differ in their responses to external N enrichment because they rely on contrasting nitrogen-acquisition strategies. Specifically, we expected *Azolla* to maintain a relative advantage under low-N conditions due to symbiotic N fixation, whereas *Lemna* would become increasingly competitive with increasing N availability because of its stronger dependence on dissolved inorganic nitrogen and greater growth plasticity. We further hypothesized that this shift in competitive balance would be accompanied by coordinated changes in root traits and elemental stoichiometry. Specifically, we aimed to (1) quantify how N enrichment and differences between the two experimental periods were associated with variation in root traits and C:N:P stoichiometry; (2) determine how these functional adjustments were associated with competitive balance and potential submerged niche partitioning; and (3) identify the N level or approximate threshold range at which community dominance shifts from *Azolla* to *Lemna*. These questions are important because they help explain how nitrogen management may influence whether floating-plant communities in paddy systems are more strongly dominated by *Azolla* or by *Lemna*.

## 2. Results

### 2.1. Plant Growth and Biomass Accumulation

**Figure 1:**
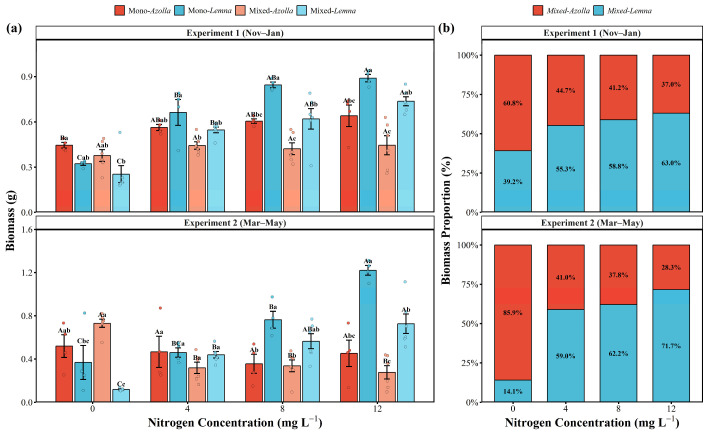
Biomass and biomass proportion of *Azolla* and *Lemna* under different nitrogen concentrations: (**a**) Biomass of *Azolla* and *Lemna* under monoculture and mixed-culture treatments. (**b**) Biomass proportion of *Azolla* and *Lemna* in the mixed-culture treatment. Notes: The upper and lower panels in both (**a**,**b**) represent Experiment 1 (Nov–Jan) and Experiment 2 (Mar–May), respectively. Values are presented as mean ± SE, with individual replicate points overlaid in panel (**a**). Sample sizes were n = 4 for each monoculture treatment and n = 6 for each mixed-culture treatment in each experimental period. Different lowercase letters indicate significant differences among treatment groups at the same nitrogen level (*p* < 0.05), and different uppercase letters indicate significant differences among nitrogen levels within the same treatment group (*p* < 0.05).

ANOVA revealed that culture mode had a significant main effect on the biomass of both *Azolla* and *Lemna* (*p* < 0.01, Table 1). While nitrogen (N) concentration had a highly significant main effect on the biomass of *Lemna* (*p* < 0.001), significant N × culture interactions were observed for both species (*p* < 0.05, Table 1). Across both experimental periods, the biomass of both species generally increased with increasing N concentration, although the response was much stronger in *Lemna* (Figure 1a). Under N-deficient conditions (0 mg L^−1^), *Azolla* produced more biomass than *Lemna* in both monoculture and mixed cultures. However, *Lemna* exhibited a steeper growth response to N addition; at high N levels (8 and 12 mg L^−1^), its biomass increased sharply, reaching levels comparable to or higher than those of *Azolla* in mixed treatments (Figure 1a). Accordingly, biomass proportions in mixed cultures showed a clear N-driven shift in dominance (Figure 1b). *Azolla* dominated at 0 mg L^−1^ N, accounting for up to 60.8–90.4% of the community biomass. Conversely, *Lemna* progressively became dominant with increasing N supply, with its biomass proportion rising to 63.0–71.7% at 12 mg L^−1^ N (Figure 1b). This pattern indicates that nitrogen enrichment was associated with changes not only in plant growth but also in the relative dominance of the two species in mixed culture, shifting community structure from *Azolla*-dominated to *Lemna*-dominated pattern.

### 2.2. Root Morphological Traits

**Figure 2:**
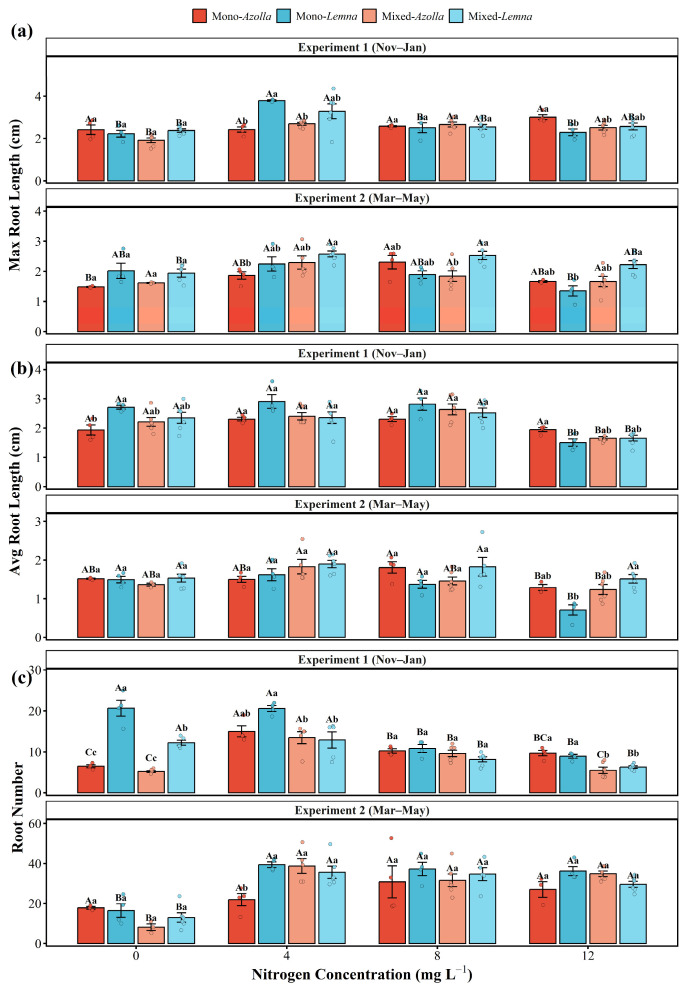
Root morphological traits of *Azolla* and *Lemna* under different nitrogen levels and culture modes. Notes: The traits include (**a**) maximum root length, (**b**) average root length, and (**c**) root number. In each subplot, the upper and lower panels represent Experiment 1 (Nov–Jan) and Experiment 2 (Mar–May), respectively. Values are presented as mean ± SE, with individual replicate points overlaid. Sample sizes were n = 4 for each monoculture treatment and n = 6 for each mixed-culture treatment in each experimental period. Uppercase letters indicate significant differences among N levels within the same treatment group, whereas lowercase letters indicate significant differences among treatment groups at the same N level (*p* < 0.05).

ANOVA indicated that both nitrogen (N) concentration and experimental period had highly significant main effects on all evaluated root morphological traits (maximum root length, average root length, and root number) for both *Azolla* and *Lemna* (*p* < 0.001, Table 1). Culture mode significantly affected the maximum root length and root number of *Lemna* but had no significant main effect on any root trait of *Azolla*. Furthermore, significant N × culture interactions were observed for the maximum root length and root number of *Azolla*, as well as the average root length of *Lemna* (*p* < 0.05, Table 1). Across both species, root length generally peaked at low-to-moderate N levels (4 or 8 mg L^−1^) before declining at the highest N concentration (12 mg L^−1^) (Figure 2). Clear differences between the two experimental periods were also observed in root traits: plants in Experiment 1 generally developed longer maximum and average roots (Figure 2a,b), whereas plants in Experiment 2 produced more roots (Figure 2c). Compared with monocultured *Lemna*, plants grown in mixed culture generally produced fewer roots but developed longer maximum roots (particularly in Experiment 2) across the N gradients (Figure 2a,c).

### 2.3. Plant Ecological Stoichiometry

**Figure 3:**
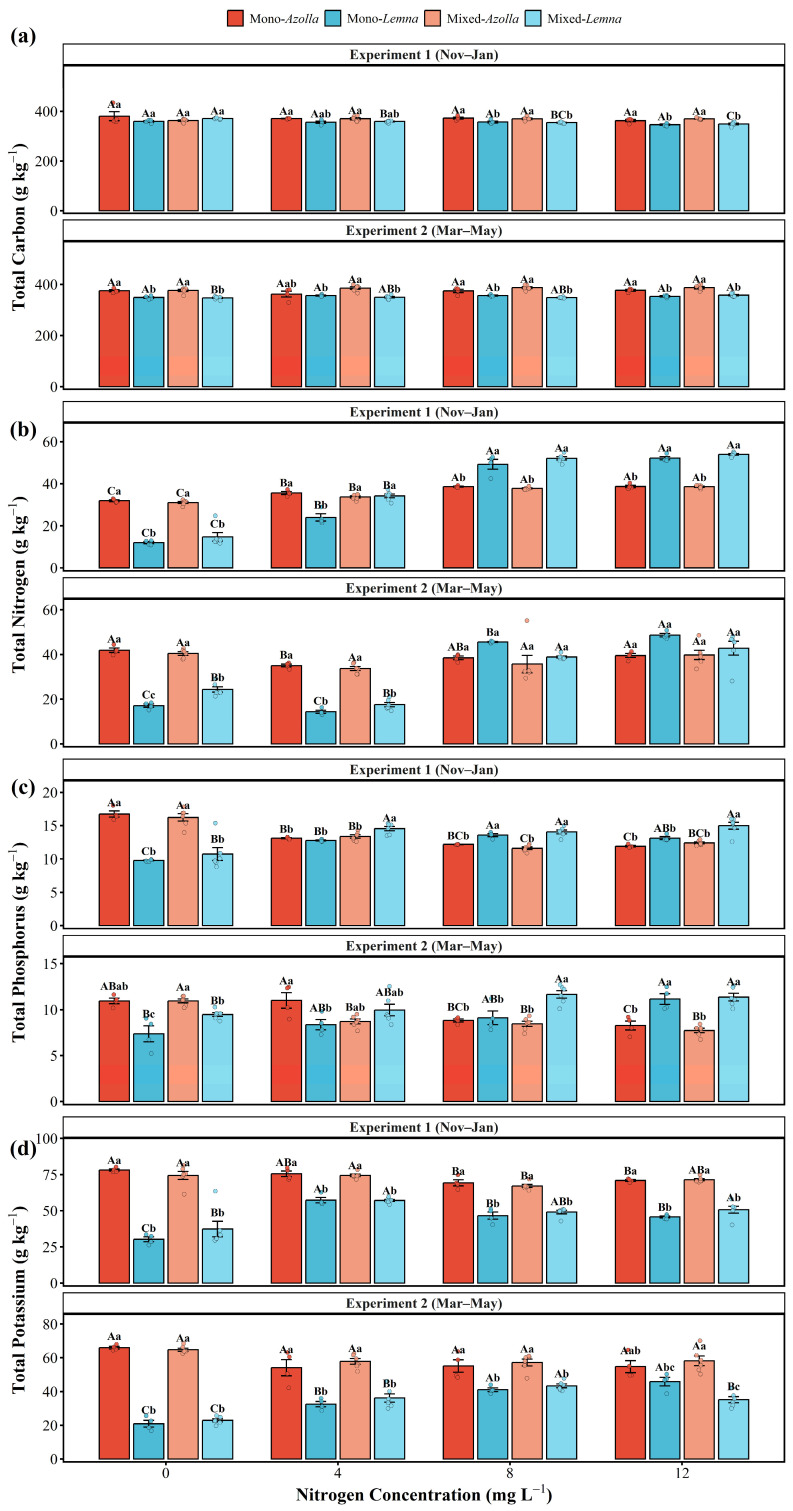
Elemental contents of *Azolla* and *Lemna* under different nitrogen levels and culture modes: (**a**) total carbon (TC); (**b**) total nitrogen (TN); (**c**) total phosphorus (TP); (**d**) total potassium (TK). Notes: The upper and lower panels in each subplot represent Experiment 1 (Nov–Jan) and Experiment 2 (Mar–May), respectively. Values are presented as mean ± SE, with individual replicate points overlaid. Sample sizes were n = 4 for each monoculture treatment and n = 6 for each mixed-culture treatment in each experimental period. Different lowercase letters indicate significant differences among treatment groups at the same nitrogen level (*p* < 0.05), and different uppercase letters indicate significant differences among nitrogen levels within the same treatment group (*p* < 0.05).

ANOVA revealed that nitrogen concentration significantly affected most elemental contents and stoichiometric ratios in both species, although TC was insensitive to N in *Azolla* (*p* < 0.05, Table 1). Across both experimental periods, TC content varied less than the other elemental traits, although experimental-period effects were significant in both species, and a modest N effect was detected in *Lemna* (Figure 3a; Table 1). TN content increased with N supply in both species, with a much stronger response in *Lemna*. By contrast, TK content showed species-specific patterns, increasing in *Lemna* but tending to decline in *Azolla*. The progressive accumulation of TN was particularly pronounced in *Lemna* across the entire N gradients in both experimental periods (Figure 3b). Similarly, TK contents peaked at moderate-to-high N levels (Figure 3d). In contrast, *Azolla* exhibited an opposing elemental response: while its TN content showed a moderate overall increase with N addition, its TP content exhibited a distinct decreasing trend. A similar overall declining pattern was observed for its TK content, although this declining trend was less pronounced in Experiment 2 (Figure 3c,d). Notably, the stronger accumulation of TN and TP in *Lemna* under elevated N was accompanied by its sharper biomass increase and increasing dominance in mixed culture, whereas the more moderate elemental shifts in *Azolla* were consistent with its comparatively stable growth response across the N gradient.

### 2.4. Plant Stoichiometric Ratios

ANOVA indicated that nitrogen (N) concentration had a highly significant main effect on both the carbon-to-nitrogen (C:N) and nitrogen-to-phosphorus (N:P) ratios of *Azolla* and *Lemna* (*p* < 0.001, Table 1). The experimental period significantly influenced the N:P ratios of both species (*p* < 0.001) but had no significant effect on their C:N ratios. Additionally, culture mode significantly affected the C:N ratios of both species and the N:P ratio of *Lemna*. Significant N × culture interactions were observed for both the C:N and N:P ratios of *Lemna* (*p* < 0.001, Table 1).

**Figure 4:**
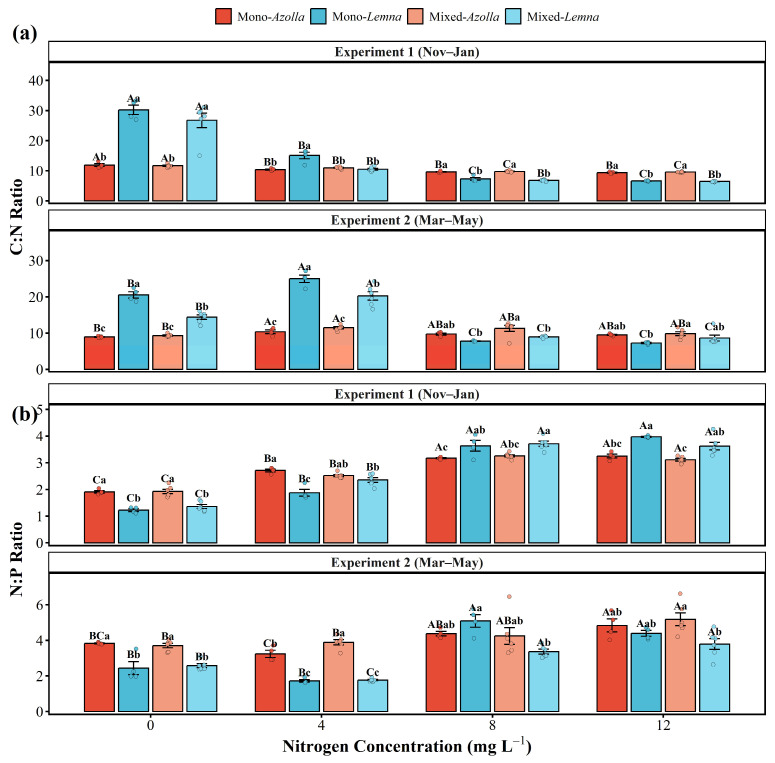
Stoichiometric ratios of *Azolla* and *Lemna* under different nitrogen levels and culture modes: (**a**) C:N ratio and (**b**) N:P ratio. Notes: The upper and lower panels in each subplot represent Experiment 1 (Nov–Jan) and Experiment 2 (Mar–May), respectively. Values are presented as mean ± SE, with individual replicate points overlaid. Sample sizes were n = 4 for each monoculture treatment and n = 6 for each mixed-culture treatment in each experimental period. Different lowercase letters indicate significant differences among treatment groups at the same nitrogen level (*p* < 0.05), and different uppercase letters indicate significant differences among nitrogen levels within the same treatment group (*p* < 0.05).

Across the N gradients, the stoichiometric ratios of the two species exhibited different response patterns (Figure 4). As N concentration increased from 0 to 12 mg L^−1^, the C:N ratio of *Lemna* decreased substantially. By contrast, the C:N ratio of *Azolla* showed a slight decrease and remained within a relatively narrow range across all N treatments (Figure 4a). Conversely, the N:P ratios of both species showed an overall upward trend with increasing N supply (Figure 4b). Consistent with the significant interactive effects, the stoichiometric ratios of *Lemna* showed clear culture-dependent differences across the N gradient, with especially clear differences observed at 0 mg L^−1^ for the C:N ratio and at higher N levels (e.g., 8 and 12 mg L^−1^) for the N:P ratio (Figure 4). Together, these stoichiometric patterns paralleled the phenotypic divergence between the two species: the marked decline in C:N and stronger rise in N:P in *Lemna* under high N were accompanied by greater biomass gain and a shift toward dominance in mixed culture, whereas the narrower stoichiometric range of *Azolla* corresponded to its more conservative growth pattern and relatively stable root expression.

### 2.5. Interspecific Competition and Relative Yields

**Figure 5:**
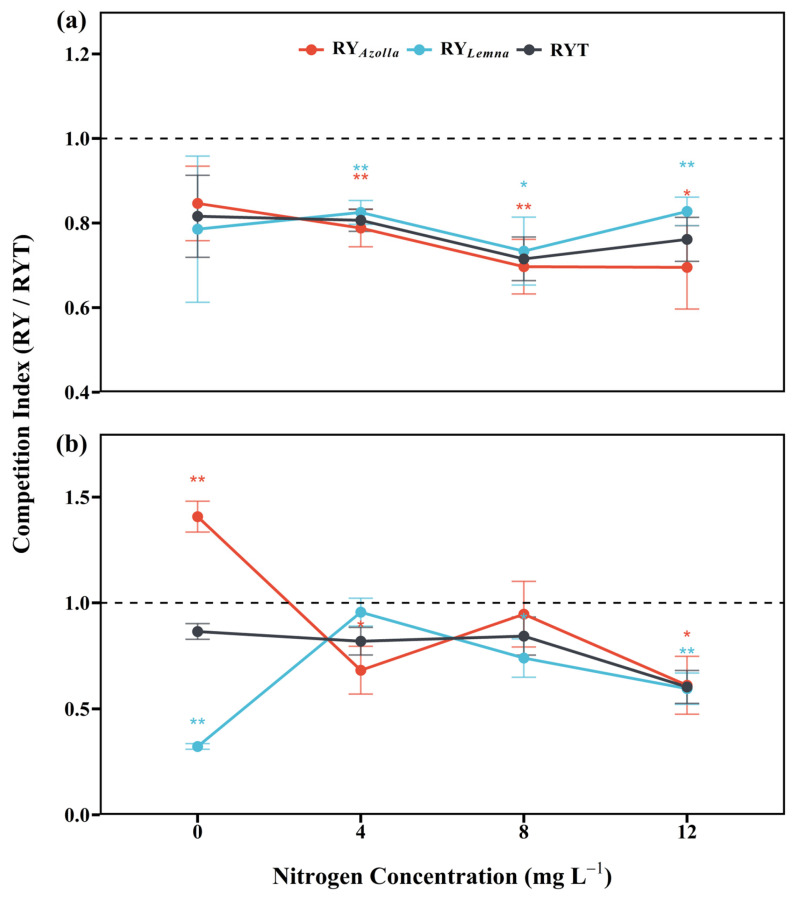
Interspecific competitive interactions between *Azolla* and *Lemna* across a nitrogen gradient. Notes: (**a**) Experiment 1 (November–January). (**b**) Experiment 2 (March–May). Interspecific competition was evaluated using the Relative Yield (RY) of each species and the Relative Yield Total (RYT) of the mixed system. Values are presented as mean ± SE. The dashed horizontal line at 1.0 represents the threshold for neutral competition. Asterisks indicate significant deviations from the neutral threshold (1.0) based on one-sample *t*-tests (* *p* < 0.05, ** *p* < 0.01). Sample sizes for species-level RY were n = 6 per treatment in each experimental period.

Interspecific competition between *Azolla* and *Lemna* varied with nitrogen (N) availability (Figure 5). Across both experimental periods, a consistent trend of stronger competition was observed as N supply increased. At the highest N concentration (12 mg L^−1^), the relative yields (RY) of both *Azolla* and *Lemna* were significantly lower than 1.0 (*p* < 0.05) (Figure 5a,b). Correspondingly, the Relative Yield Total (RYT) was generally below 1.0 and tended to decline with increasing N supply. Conversely, under N-deficient conditions (0 mg L^−1^), competitive performance shifted in favor of *Azolla*. This was most prominent in experiment 2, where the RY of *Azolla* at 0 mg L^−1^ reached 1.41 (significantly > 1.0, *p* < 0.01), while *Lemna* was strongly suppressed (RY = 0.32, *p* < 0.01) (Figure 5b). Differences between the two experimental periods were also evident, suggesting that competitive outcomes were associated not only with external N supply but also with variation between the two experimental runs. Consistent with this pattern, the experimental period significantly affected several root morphological traits and nutrient-related traits in both species (Table 1; Figure 2, Figure 3 and Figure 4), indicating that variation in trait expression between the two experimental periods may have contributed to the observed differences in competition intensity.

### 2.6. Species-Specific Phenotypic Plasticity and Contrasting Adaptive Strategies

**Figure 6:**
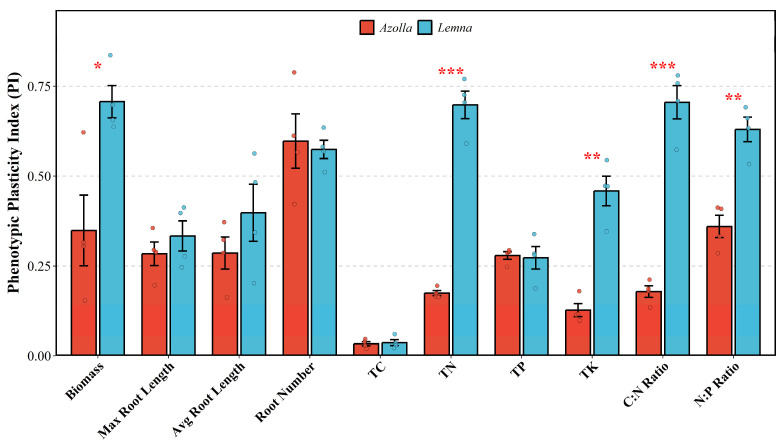
Phenotypic plasticity index (PI) of *Azolla* and *Lemna* for different functional traits under the nitrogen gradient. Notes: Bars represent mean PI values for *Azolla* and *Lemna*, with error bars indicating SE. Sample sizes were n = 4 per species for each trait (2 experiments × 2 cultivation modes). Significant interspecific differences in PI for each trait were tested using independent *t*-tests and are indicated by asterisks (* *p* < 0.05, ** *p* < 0.01, *** *p* < 0.001).

Our plasticity index (PI) analysis revealed significant interspecific differences in phenotypic responses to the N gradients (Figure 6). Specifically, for biomass, *Lemna* demonstrated high plasticity (PI = 0.71), whereas *Azolla* showed lower plasticity in biomass (PI = 0.35). At the elemental level, *Lemna* showed marked plasticity in response to TN (PI = 0.70) and TK (PI = 0.46). Conversely, *Azolla* showed relatively low plasticity in response to elemental composition, particularly for TN (PI = 0.17) and TC (PI = 0.03). Similarly, ecological stoichiometry reflected this divergence: the C:N ratio of *Lemna* was highly plastic (PI = 0.71), whereas *Azolla* exhibited lower stoichiometric plasticity (PI = 0.18 for C:N ratio), with the N:P ratio following a similar pattern.

### 2.7. Multivariate Trait Coordination

**Figure 7:**
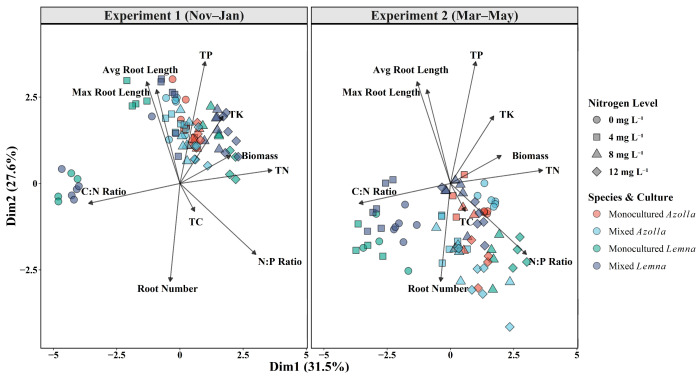
Principal component analysis (PCA) biplot illustrating the multivariate trait coordination of *Azolla* and *Lemna* across nitrogen gradients and experimental periods. Notes: The biplot displays both individual sample points and trait vectors (arrows) within a unified global PCA space. To facilitate comparison, samples are distinctly faceted into Experiment 1 (Nov–Jan; (**left**) panel) and Experiment 2 (Mar–May; (**right**) panel). Point colors denote species and culture modes: monocultured *Azolla* (Mono-A, red), mixed-culture *Azolla* (Mixed-A, light blue), monocultured *Lemna* (Mono-L, green), and mixed-culture *Lemna* (Mixed-L, dark blue). Point shapes represent the nitrogen concentration levels (0–12 mg L^−1^).

A global principal component analysis (PCA) was employed to evaluate multivariate trait responses across N gradients and experimental periods (Figure 7). The first two principal components (Dim1 and Dim2) jointly explained 59.1% of the total variance. Dim1 (31.5%) was strongly associated with N-related trait variation. In both panels, trait vectors for total nitrogen (TN) and biomass pointed toward the positive direction of Dim1, whereas the C:N ratio pointed toward the negative direction. Accordingly, sample points tended to shift from left to right along Dim1 as N supply increased (Figure 7). Furthermore, the second principal component (Dim2, 27.6%) captured clear differences between the two experimental periods in trait coordination. Data points from Experiment 1 (Nov–Jan) were mainly distributed in the positive region of Dim2, tightly aligning with the vectors for Max Root Length, Avg Root Length, TP, and TK. Conversely, samples from Experiment 2 (Mar–May) mainly clustered in the negative lower half and were more closely associated with Root Number and N:P ratio. Together, these patterns suggest that both nitrogen availability and differences between the two experimental periods were associated with trait coordination, but at different levels: N supply primarily structured the main growth–stoichiometry gradient along Dim1, whereas differences between the two experimental periods were more strongly associated with variation in trait combinations along Dim2. Thus, the observed PCA clustering indicates that plant responses were jointly associated with N availability and variation between the two experimental periods.

## 3. Discussion

### 3.1. Nitrogen Availability Shifts Competitive Dominance Through Divergent Growth Strategies

The competitive outcomes between *Azolla* and *Lemna* were closely associated with N availability, reflecting contrasting positions along a conservative–acquisitive strategy axis [23,24]. Under N-deficient conditions (0 mg L^−1^), *Azolla* maintained clear competitive dominance, accounting for over 60.8–90.4% of the biomass in mixed cultures. This advantage is likely attributable to its symbiotic relationship with the nitrogen-fixing cyanobacterium, which allows *Azolla* to reduce its dependence on external N supply. In the context of the plant economics spectrum, *Azolla* exhibited a ‘slower’ resource-conservative strategy, characterized by strict stoichiometric homeostasis and steady growth when environmental N was scarce. Similarly, long-term experimental evidence from unproductive grasslands suggests that dominance by slow-growing, long-lived species confers a remarkable ‘stabilizing capacity’ to the community, allowing it to resist drastic compositional shifts despite environmental fluctuations [25]. Furthermore, our findings align with recent studies on aquatic plant community dynamics, which demonstrate that stress-tolerant species (such as submerged macrophytes) often dominate under low nutrient regimes but are progressively replaced by more aggressive competitors as nitrogen levels increase [26].

However, as external N concentration increased, the competitive balance shifted markedly. The significant N × culture interaction indicates that, in both experiments, the growth of *Lemna* was highly responsive to nutrient enrichment under mixed-culture conditions. Relieved from N limitation, *Lemna* shifted toward the ‘fast’ end of the economics spectrum, adopting an opportunistic ‘acquisitive’ strategy. By contrast, *Azolla* may incur additional metabolic costs associated with maintaining diazotrophic symbionts, which could constrain its growth response under high-N conditions. Consequently, under high N conditions (12 mg L^−1^), *Lemna* increased rapidly and likely occupied more surface space, which may have reduced light availability for *Azolla*. The shift in biomass proportion in mixed culture further indicates that nitrogen enrichment changed the relative dominance of the two species, rather than simply increasing total plant biomass. Overall, these results suggest that increasing N availability was associated with a shift in floating-plant communities from *Azolla* dominance toward *Lemna* dominance, consistent with the advantage of faster and more plastic growth strategies under N-rich conditions [25,27].

### 3.2. Species-Specific Root Foraging

Root morphological plasticity reflects the trade-off between nutrient acquisition and carbon allocation. In free-floating macrophyte communities, species with more responsive root traits, such as changes in root length and root number, may show stronger nutrient uptake and faster growth under nutrient-rich conditions [28]. In our study, *Lemna* exhibited a highly plastic, hump-shaped root response to nitrogen. Root expansion under low-to-moderate N is consistent with a potential foraging response that may enhance resource acquisition when nutrients are limiting. However, because we did not directly measure nutrient uptake position, root surface area, root biomass, or root uptake rates, this pattern should be interpreted cautiously as a trait response rather than direct evidence of a specific uptake mechanism [29]. The suppression of root development at the highest N level (12 mg L^−1^) suggests that when nitrogen is no longer limiting, *Lemna* may reallocate carbon from roots toward frond growth. This interpretation is broadly consistent with studies showing that N enrichment often reduces root investment while increasing allocation to aboveground growth [30]. By contrast, *Azolla* maintained relatively stable root architecture across the N gradients. Partly supported by symbiotic N fixation, *Azolla* may be less dependent on continual root restructuring, a pattern consistent with a more resource-conservative strategy [23].

Clear differences between the two experimental periods also influenced root development. Background environmental differences between the two periods, including temperature, may have contributed to this contrast [31]. One possible explanation is that the relatively lower temperatures during the first period were associated with greater root elongation, whereas the relatively warmer conditions during the second period were associated with greater root proliferation. These contrasts are consistent with previous work showing that temperature fluctuations can alter trait expression and interspecific interactions in aquatic macrophyte communities [32].

A particularly interesting finding is the morphological adjustment of *Lemna* when co-cultured with *Azolla*. Under interspecific competition, *Lemna* generally produced fewer but longer roots than in monoculture, especially in Experiment 2 and under higher N levels. This pattern may reflect a shift in root morphology in response to interspecific competition [33]. Because elongated roots require additional carbon investment, the concurrent reduction in root number suggests a compensatory trade-off that may allow *Lemna* to extend its rooting zone while limiting the carbon costs of maintaining numerous roots.

The marked difference between the two experimental periods in competition indices further suggests that nitrogen availability alone did not determine competitive outcomes. Instead, differences between the two experimental runs were associated with overall plant performance and trait expression. In our experiment, the experimental period significantly affected several root morphological traits and nutrient-related traits, and the PCA also showed clear separation between the two experimental runs in multivariate trait coordination. Because direct nitrogen uptake rates were not measured, we cannot conclude that differences between the two experimental periods directly changed N uptake per se. However, the observed differences between the two experimental periods in root traits, elemental contents, and stoichiometric ratios indicate that temporal context was associated with variation in the intensity and direction of interspecific competition.

### 3.3. Stoichiometric Homeostasis Versus Luxury Consumption

Ecological stoichiometry provides a powerful framework to interpret the divergent life-history strategies of floating macrophytes [14,15,34]. In our study, *Azolla* exhibited comparatively stronger stoichiometric homeostasis than *Lemna*. Although its TN content increased and its C:N ratio declined with N addition, these changes were much smaller than those observed in *Lemna*. In addition, the declines in TP and TK contents under high N suggest tighter nutrient regulation and limited luxury consumption in *Azolla*. This pattern may help maintain elemental balance within the *Azolla*–cyanobiont symbiosis while reducing unnecessary metabolic costs associated with excess nutrient accumulation [35,36].

By contrast, *Lemna* displayed high stoichiometric flexibility. Consistent with previous studies identifying nitrogen as a major driver of duckweed proliferation [37], *Lemna* accumulated more TN, TP, and TK as external N increased, accompanied by a sharp decline in its C:N ratio. This pattern is consistent with luxury nutrient uptake, an acquisitive strategy commonly observed in fast-growing plants under nutrient-rich conditions. Although *Lemna* also increased P accumulation under high N supply, the stronger increase in N resulted in an overall rise in its N:P ratio.

Differences between the two experimental periods further differentiated the responses of C:N and N:P. The C:N ratio was not significantly affected by the experimental period in either species, suggesting that the balance between carbon investment and nitrogen assimilation was relatively stable across the two experimental periods. By contrast, the N:P ratio responded strongly to differences between the two experimental periods. This pattern is broadly consistent with the growth rate hypothesis (GRH), which predicts greater P demand under conditions favoring rapid growth. In our study, the observed difference in N:P ratio between the two experimental periods may reflect broader differences in trait expression and nutrient demand between the two runs. Background environmental differences, potentially including temperature, may have contributed to this pattern, although this interpretation remains tentative because related physiological variables were not directly measured [38]. This interpretation is also supported by the PCA, which showed that nitrogen availability and differences between the two experimental periods were associated with trait variation in complementary ways. The primary axis was mainly associated with biomass, TN, and C:N ratio, suggesting that external N supply was associated with the major gradient of growth and nutrient accumulation. By contrast, the second axis separated the experimental runs and was more strongly associated with root morphology and nutrient-balance traits, suggesting that differences between the two experimental periods were associated with differences in how plants expressed their trait combinations under a given N level. Overall, nitrogen availability was associated with the direction of species responses, whereas variation between the two experimental periods was associated with the trait configuration through which those responses were expressed.

### 3.4. Ecological Implications for Sustainable Paddy Field Management

The contrasting strategies of *Azolla* and *Lemna* provide useful insights for nutrient management in paddy ecosystems. The conservative growth pattern and N-fixing capacity of *Azolla* suggest that it is particularly suitable for low-input or organic systems, especially under low-N conditions where its competitive advantage can be maintained [37,38]. By contrast, the high nutrient uptake capacity of *Lemna* may be beneficial in nutrient-rich systems for rapid nutrient removal [39]. Rather than indicating fully interchangeable roles, these differences suggest a degree of functional complementarity between the two macrophytes. *Lemna* may respond quickly to short-term nutrient enrichment, whereas *Azolla* may contribute more to biological N inputs and nutrient retention under low-N conditions. More broadly, the contrasting root plasticity of *Lemna* and the stronger stoichiometric homeostasis of *Azolla* suggest that floating macrophyte assemblages may help regulate paddy-water nutrient dynamics under varying environmental conditions.

This study also has several limitations. The relationships between trait variation and competitive outcomes are correlative rather than directly mechanistic. In addition, because the two experimental periods were included only as an additive factor in the statistical model, differences between them should be interpreted cautiously as variation between the two experimental runs. Furthermore, the concept of an “individual” was not strictly equivalent between *Azolla* and *Lemna*. Although the initial number of units was standardized, initial biomass and surface area were not measured, which may have influenced the magnitude of the observed competitive differences.

## 4. Materials and Methods

### 4.1. Study Site and Materials

The experiment was conducted in a greenhouse at South China Agricultural University, Guangzhou, China (23°16′ N, 113°36′ E). *Azolla filiculoides* (hereafter *Azolla*) and *Lemna minor* (hereafter *Lemna*) were obtained from local commercial suppliers. Before the formal experiment, both species were pre-cultivated in the greenhouse for two weeks in the same nutrient solution to allow acclimation. Healthy individuals of similar size and normal pigmentation were then selected for the experiment to minimize initial physiological variation.

### 4.2. Experimental Design

The experiment was conducted in two experimental runs: the first from November 2022 to January 2023 and the second from March to May 2023. For descriptive purposes, these two runs are referred to as the first and second experimental periods throughout the manuscript. For each run, a two-factor factorial design was used, including three cultivation modes (*Azolla* monoculture, *Lemna* monoculture, and *Azolla* + *Lemna* mixed culture) and four exogenous nitrogen concentrations (0, 4, 8, and 12 mg N L^−1^, hereafter N0, N4, N8, and N12, respectively). In general, concentrations below 12 mg N L^−1^ are representative of paddy-water N levels during much of the flooding period, whereas 12 mg N L^−1^ represents a relatively enriched condition that may occur transiently following fertilization. For analysis and figure presentation, responses in the mixed-culture treatment were separated by species, resulting in four treatment groups: Mono-*Azolla*, Mono-*Lemna*, Mixed-*Azolla*, and Mixed-*Lemna*. Four biological replicates were used for each monoculture treatment and six replicates for each mixed-culture treatment, giving a total of 56 experimental units per experimental period.

All plants were cultivated in 5 L cylindrical buckets, providing a consistent physical water surface area to standardize spatial competition across all treatments. Because *Azolla* and *Lemna* differ in morphology and clonal organization, initial standardization was based on individual counts rather than directly equivalent initial biomass or surface area. In the monoculture treatments, 15 healthy individuals were introduced into each microcosm. In the mixed-culture treatment, an additive design was adopted, with 15 individuals of *Azolla* and 15 individuals of *Lemna* co-inoculated into each bucket. This design enabled comparison of species performance in the presence and absence of interspecific competition.

All treatments were maintained in a greenhouse under natural light. Deionized water and a 10-fold diluted modified N-free Hoagland solution were used as the basal medium to provide essential nutrients while avoiding additional N input, and Ca was supplied by adding 10% CaCl_2_ solution [40]. The composition of the modified Hoagland solution is shown in Table 2. To simulate agriculturally relevant nitrogen inputs, urea—the predominant synthetic fertilizer used in paddy systems—was selected as the sole nitrogen source. During the experiment, no additional nutrients or N sources were added. Water lost through evaporation was replenished regularly with distilled water to maintain a similar water level among buckets.

### 4.3. Trait Measurements

At the end of each experimental run, plants from each bucket were harvested separately by species. Before harvest, intact individuals were randomly selected from each bucket for root trait measurements. For each selected individual, maximum root length, average root length, and root number were determined using a digital caliper and manual counting. After root measurements, all biomass of each species in each bucket was collected, rinsed with deionized water, and gently blotted dry with absorbent paper. Samples were first oven-dried at 105 °C for 30 min to stop enzymatic activity and then dried at 75 °C to constant weight. Dry biomass was recorded using an analytical balance with a precision of 0.001 g.

Elemental analyses followed standard protocols described in previous studies [41]. The dried samples were homogenized, ground into a fine powder, and passed through a 100-mesh sieve for elemental analysis. Total carbon (TC) and total nitrogen (TN) contents were determined using an elemental analyzer. For total phosphorus (TP) and total potassium (TK), samples were digested with an H_2_SO_4_–H_2_O_2_ mixture. TP content was determined by the molybdenum–antimony colorimetric method, and TK content was measured using inductively coupled plasma optical emission spectrometry (ICP-OES). Stoichiometric ratios (C:N and N:P) were then calculated based on the measured elemental concentrations.

### 4.4. Statistical Analysis

All data are presented as mean ± standard error (SE), unless otherwise stated. All statistical analyses and graphical visualizations were performed in R software (version 4.3.2), and figures were generated using the ggplot2 package. Prior to analysis, the normality of residuals and homogeneity of variances were evaluated using the Shapiro–Wilk test and Levene’s test, respectively. Differences were considered statistically significant at *p* < 0.05.

For biomass, root morphological traits, elemental contents, and stoichiometric ratios, the data of *Azolla* and *Lemna* were analyzed separately. For each response variable, analysis of variance (ANOVA) was used to test the effects of nitrogen level (N), culture mode (C), experimental period (E), and the N × C interaction, using the following model:

Response ~ N × C + E

where N level, culture mode, and experimental period were treated as fixed factors. Because the experimental period was included as an additive factor in the fitted model, interactions involving the experimental period were not tested.

To examine differences among the four N levels within each species–culture combination, one-way ANOVA followed by Tukey’s Honestly Significant Difference (HSD) test was performed. Comparisons between monoculture and mixed culture within the same N level were conducted using one-way ANOVA.

To quantify interspecific competition, relative yield (RY) and relative yield total (RYT) were calculated as

RY = Y_mix_/Y_mono_

RYT = RY*_Azolla_* + RY*_Lemna_*

where Y_mix_ and Y_mono_ represent the biomass of a given species in mixed culture and monoculture, respectively. One-sample *t*-tests were used to determine whether RY and RYT differed significantly from the neutral expectation of 1.0.

Phenotypic plasticity was quantified using the plasticity index (PI), calculated as

PI = (Max − Min)/Max

where Max and Min are the maximum and minimum mean values of a given trait across the four N levels within each Experimental period × Species × Culture combination. For each trait, mean PI values were then calculated for *Azolla* and *Lemna*, and interspecific differences in PI were tested using independent-samples *t*-tests.

A global principal component analysis (PCA) was conducted to evaluate multivariate trait coordination and overall trait variation across nitrogen levels, culture modes, and experimental periods. PCA was performed using the FactoMineR package, and the biplots were visualized using the factoextra package to display both sample distributions and trait vectors in a common ordination space. To facilitate comparison between the two experimental periods, PCA scores were presented separately for each experimental period.

## 5. Conclusions

In this microcosm experiment, which combined four nitrogen levels with monoculture and mixed-culture treatments across two experimental periods, *Azolla* and *Lemna* showed contrasting growth and nutrient-use strategies. In mixed culture, *Azolla* maintained a competitive advantage under low-N conditions, whereas *Lemna* became progressively dominant as N availability increased. These shifts in relative dominance were associated with stronger root plasticity and greater stoichiometric flexibility in *Lemna*, whereas *Azolla* showed more stable root expression and stronger stoichiometric homeostasis. Overall, the results indicate that nitrogen enrichment is associated with changes in the competitive balance between these two floating macrophytes, with implications for the management of *Azolla*-dominated versus *Lemna*-dominated communities in paddy ecosystems.

## Figures and Tables

**Table 1:** ANOVA results for the effects of nitrogen level, culture mode, their interaction, and experimental period on the growth and stoichiometric traits of *Azolla* and *Lemna*.

Species	Trait	N Effect	C Effect	E Effect	N × C
*Azolla*	Biomass	2.32 ns	7.26 **	2.44 ns	2.97 *
*Azolla*	Max root length	10.13 ***	1.32 ns	84.36 ***	3.93 *
*Azolla*	Avg root length	15.30 ***	0.21 ns	106.97 ***	1.56 ns
*Azolla*	Root number	14.52 ***	0.94 ns	197.18 ***	3.13 *
*Azolla*	TC	0.35 ns	2.09 ns	11.50 **	2.28 ns
*Azolla*	TN	7.51 ***	2.28 ns	9.79 **	0.31 ns
*Azolla*	TP	98.42 ***	6.51 *	602.11 ***	1.52 ns
*Azolla*	TK	11.02 ***	0.03 ns	161.52 ***	0.80 ns
*Azolla*	C:N	8.72 ***	7.48 **	2.80 ns	1.14 ns
*Azolla*	N:P	30.19 ***	0.35 ns	187.04 ***	0.36 ns
*Lemna*	Biomass	64.70 ***	34.81 ***	1.15 ns	2.83 *
*Lemna*	Max root length	15.94 ***	6.07 *	36.28 ***	2.41 ns
*Lemna*	Avg root length	22.55 ***	0.61 ns	104.12 ***	3.54 *
*Lemna*	Root number	23.71 ***	20.04 ***	299.62 ***	0.51 ns
*Lemna*	TC	3.08 *	0.11 ns	16.14 ***	3.34 *
*Lemna*	TN	496.39 ***	7.01 *	67.07 ***	9.73 ***
*Lemna*	TP	29.63 ***	28.58 ***	137.38 ***	0.27 ns
*Lemna*	TK	51.87 ***	1.52 ns	112.79 ***	1.7 ns
*Lemna*	C:N	223.84 ***	17.48 ***	0.59 ns	8.64 ***
*Lemna*	N:P	175.89 ***	6.84 *	17.41 ***	8.80 ***

Note: Values represent *F*-statistics. N effect: main effect of nitrogen concentration; C effect: main effect of culture mode (monoculture vs. mixed culture); E effect: main effect of experimental period (Experiment 1 vs. Experiment 2); N × C: interaction effect between nitrogen concentration and culture mode. In the fitted ANOVA model, the experimental period was included as an additive factor, and interactions involving the experimental period were not tested. Significance levels are indicated as follows: *** *p* < 0.001; ** *p* < 0.01; * *p* < 0.05; ns, not significant (*p* > 0.05).

**Table 2:** Composition of the modified N-free Hoagland solution.

Compound	Concentration (mg·L^−1^)	Compound	Concentration (mg·L^−1^)
KCl	152.828	MnSO_4_·H_2_O	0.338
K_2_HPO_4_·3H_2_O	456.638	CuSO_4_	0.125
MgSO_4_·7H_2_O	246.48	ZnSO_4_·H_2_O	0.576
NaFeC_10_H_12_N_2_O_8_	11.17	Na_2_MoO_4_	0.102
H_3_BO_3_	1.546		

## Data Availability

The data presented in this study are available on request from the corresponding author. The data are not publicly available due to privacy restriction.
